# Optimization on Preparation Conditions of Salidroside Liposome and Its Immunological Activity on PCV-2 in Mice

**DOI:** 10.1155/2015/178128

**Published:** 2015-03-23

**Authors:** Yibo Feng, Xiaojuan Zhao, Fang Lv, Jinqiu Zhang, Bihua Deng, Yanhong Zhao, Yuanliang Hu, Deyun Wang, Jiaguo Liu, Yu Lu, Ruonan Bo, Zhenguang Liu

**Affiliations:** ^1^Institute of Traditional Chinese Veterinary Medicine, College of Veterinary Medicine, Nanjing Agricultural University, Nanjing 210095, China; ^2^National Research Center of Veterinary Biologicals Engineering and Technology, Jiangsu Academy of Agricultural Science, Nanjing 210014, China

## Abstract

The aim of this study was to optimize the preparation conditions of salidroside liposome with high encapsulation efficiency (EE) and to study the immunological enhancement activity of salidroside liposome as porcine circovirus type 2 virus (PCV-2) vaccine adjuvant. Response surface methodology (RSM) was selected to optimize the conditions for the preparation of salidroside liposome using Design-Expert V8.0.6 software. Three kinds of salidroside liposome adjuvants were prepared to study their adjuvant activity. BALB/c mice were immunized with PCV-2 encapsulated in different kinds of salidroside liposome adjuvants. The PCV-2-specific IgG in immunized mice serum was determined with ELISA. The results showed that when the concentration of ammonium sulfate was 0.26 mol·L^−1^, ethanol volume 6.5 mL, temperature 43°C, ethanol injection rate 3 mL·min^−1^, and salidroside liposome could be prepared with high encapsulation efficiency of 94.527%. Salidroside liposome as adjuvant could rapidly induce the production of PCV-2-specific IgG and salidroside liposome I adjuvant proved to provide the best effect among the three kinds of salidroside liposome adjuvants.

## 1. Introduction

*Rhodiola rosea* is mainly distributed in the northern hemisphere Himalayas, northwest Asia and North America, growing at an altitude of 1600–4000 m alpine, dry, strong ultraviolet radiation, and large temperature region, with a strong environmental adaptability and vitality [1].* Rhodiola rosea* has been used as a herbal medicine for a long time and recorded in both* Four Medical Classics* and* Compendium of Materia Medica*. Pharmacological studies have shown that its main pharmacological active ingredient was salidroside. In recent years, it was reported that salidroside had antifatigue, antiaging, immune regulation, scavenging free radicals, enhancing memory, improving sleep and other pharmacological effects [2–4].

Liposomes are closed vesicles composed of a lipid bilayer and used as drug carriers in 1971. As drug carriers, liposomes had many superior characteristics in terms of improving the efficacy. Firstly, targeting liposomes were easy to locate in the kidney, liver, spleen, and other mononuclear phagocytes rich organs, and liposomes may be regarded as foreign substances in the body and could be engulfed by mononuclear phagocytes [5, 6]. Secondly, drugs entrapped in liposomes could be released slowly, and liposomes slowed down metabolism and excretion of drugs and prolonged action of the drug [7]. In addition, liposomes were mainly engulfed by macrophages from reticuloendothelial system, which were concentrated in the liver, spleen, and bone marrow. Compared to the free drug, drug encapsulated in liposome made drug accumulation in heart and kidney significantly lower. Thus, it may decrease the toxicity when drugs were encapsulated inliposomes [8]. Lastly, the stability of the unstable drug could be improved when it was encapsulated and protected by the liposome bilayer membrane [9].

The immunologic adjuvant properties of liposomes were first studied by Allison and Gregoriadis 40 years ago [10]. In recent years, liposomes are often employed as delivery systems for drugs or vaccine antigens to improve cellular or tissue targeting and reduce nonspecific toxicity (drug delivery) or to minimize antigen loss [11]. Thus, it was important/necessary to do some research on new formulations of salidroside and liposome to improve immunological activity and reduce dose for clinical application.

In this study, salidroside was encapsulated with liposome by ammonium sulfate gradient method and the preparation conditions were optimized by RSM method. In the optimal condition, the porcine circovirus type 2 virus (PCV-2) was encapsulated with liposome and salidroside formulation and the immunity effect of the salidroside liposome adjuvant PCV-2 vaccine was investigated. The aim of the study is to investigate whether the immunity effect of PCV-2 can be promoted after the PCV-2 was encapsulated with salidroside liposome.

## 2. Materials and Methods

### 2.1. Preparation of Salidroside Liposome

The preparation of salidroside liposome was used by ammonium sulfate gradient method. Briefly, Soybean phospholipids (Shanghai Taiwei phospholipid Ltd, China), cholesterol (Tianjin Bodi Chemical Co., Ltd, China), and Tween-80 (Shanghai Dinghao Trading Co, China) were added into ethanol at a mass ratio of 6 : 1 : 1 (w/w/w) and dissolved with appropriate ultrasound to get a stable solution. The solution was poured into ammonium sulfate solution at a constant speed. Then, the ethanol was removed by vacuum rotary evaporator. Ammonium sulfate in external aqueous phase was removed after 24 hours of dialysis in PBS (pH = 7.4). A certain concentration of salidroside (Aladdin Co., Ltd) solution was mixed with blank liposome at a certain temperature and speed for 30 min and salidroside liposome was obtained. Finally, the salidroside liposome was filtered using 0.45 *μ*m and 0.22 *μ*m millipore membrane successively.

### 2.2. The Determination of Encapsulation Efficiency

Encapsulation efficiency (EE) of salidroside liposome was measured by dialysis method [12–14]. In brief, 2 mL of salidroside liposome was added into a dialysis bag with a hydrophilic cellulose membrane (3,000 MWCO) to remove free salidroside in PBS solution (200 mL, pH = 7.4). After 24 hours of dialysis, 1 mL of salidroside liposome was placed into a 10 mL volumetric flask for analysis. 3 mL of 10% Triton X-100 (Shanghai Yuanye Biotechnology Co., Ltd, China) was used to rupture salidroside liposome. 1.5 mL of 2% sodium carbonate solution and 1 mL of azide reagent were added into the liposome solution and the solution was standing for 5 min after shaking. 5% sodium hydroxide solution was added into the volumetric flask to 10 mL. The amount of salidroside was measured by the chemical colorimetric technique utilizing reaction of diazotization in basic solution [15]. The formula to calculate the EE of salidroside liposome was shown below. “*C*_e_” was the amount of encapsulated salidroside and “*C*_t_” was the total amount of salidroside. Consider

(1) $$\begin{array}{c} \text{EE}\%=\frac{C_{\text{e}}\times 100\%}{C_{\text{t}}}. \end{array}$$

### 2.3. Optimization of Salidroside Liposome Preparation Condition

According to the previous research, four factors (ammonium sulfate concentration, ethanol injection volume, temperature, and ethanol injection rate) were the main single factors affecting the EE of salidroside liposome. On the basis of single-factor test results, four main factors were determined as follows: ammonium sulfate concentration (mol/L) (*X*_1_), ethanol injection volume (mL) (*X*_2_), temperature (°C) (*X*_3_), and ethanol injection rate (mL/min) (*X*_4_). Response surface methodology (RSM) was used to optimize the preparation conditions of salidroside liposome using Design-Expert V8.0.6 software. EE was chosen as the response (*Y*), 29 test points of a four factor-three coded level Box-Behnken design (BBD) were designed. The factors and levels were shown in Table 1.

### 2.4. Preparation of Salidroside Liposome Adjuvant PCV-2 Vaccines

Different concentrations of salidroside liposome were prepared according to the method mentioned in Section 2.1 with some modifications. Briefly, soybean phospholipids (300 mg, 600 mg, and 900 mg), cholesterol (50 mg, 100 mg, and 150 mg), and Tween-80 (50 mg, 100 mg, and 150 mg) were added into 7.0 mL of ethanol at a mass ratio of 6 : 1 : 1 (w/w/w), and the other preparation conditions remained unchanged. Three kinds of salidroside liposome were prepared and named to salidroside liposome I, salidroside liposome II, and salidroside liposome III, respectively.

Salidroside liposomes PCV-2 were prepared by repeating freeze-thaw method [16]. In brief, salidroside liposomes were mixed with PCV-2 antigen (inactivated, virus titer: 1 × 10^7.5^ TCID_50_/mL) at volume ratio of 2 : 1, 3 : 1, 4 : 1, 5 : 1, and 6 : 1. Each mixture was frozen for 3 h in −20°C. Then, they were transferred to a constant temperature oscillator for 30 min under the conditions of 45°C and 80 rpm. This operation was repeated 3 times.

#### 2.4.1. Determination of Encapsulation Efficiency of PCV-2

EE of PCV-2 was determined by BCA Protein Assay Kit (Pierce Chemical Co., USA) with some modifications [12, 13, 17]. Briefly, salidroside liposome PCV-2 was mixed with Triton X-100 at a temperature of 65°C for 10 min to release the antigen inside the liposome and was then centrifuged at 8500 g for 30 min. The protein concentration in the supernatant was measured and denoted by *C*_t_. Salidroside liposome PCV-2 without Triton X-100 was centrifuged at 8500 g for 30 min. The protein concentration in the supernatant was measured by the kit and denoted by *C*_f_. The EE of antigen was calculated according to the following formula:

(2) $$\begin{array}{c} \text{EE}=\frac{\left(C_{\text{t}}-C_{\text{f}}\right)\times 100\%}{C_{\text{t}}}. \end{array}$$

#### 2.4.2. Characteristics of Salidroside Liposome PCV-2

The particle sizes of salidroside liposome PCV-2 were measured by Hydro 2000 Mu laser particle size analyzer (Hydro2000Mu, MAL 1009117, Malvern Instruments Ltd.). The morphology of salidroside liposome PCV-2 was observed by transmission electron microscope (Tecnai 12, Holland).

#### 2.4.3. In Vitro Release Assay

In order to determine the release of PCV-2 from salidroside liposome PCV-2, 200 *μ*L of the salidroside liposome PCV-2 was mixed with 800 *μ*L of PBS (pH 7.4) and maintained at 37°C with a circulating water bath and constantly stirred by a magnetic stirrer at 150 rpm. At different time intervals, the PCV-2 concentration in PBS solution was determined using BCA Protein Assay Kit (Pierce Chemical Co., USA). The release experiments were performed in triplicate.

### 2.5. Immunological Activity of Salidroside Liposome PCV-2 in Mice

#### 2.5.1. Animals

Four-week-old BALB/c mice were purchased from Comparative Medicine Centre of Yangzhou University and acclimatized for 7 days prior to immunization. The mice were maintained under controlled conditions at temperature of 24 ± 1°C, humidity of 50 ± 10%, and a 12/12-h light–dark cycle with free access to food and water. Each mouse was used once and treated in accordance with the National Institutes of Health guide lines for the care and use of laboratory animals.

#### 2.5.2. Mouse Immunization

Mice were injected subcutaneously in the dorsal skinfold on day 0 with 0.2 mL different salidroside liposome adjuvants PCV-2 vaccines, Montanide ISA 201 adjuvant PCV-2 vaccines and PCV-2 vaccines. A booster dose was given to each primed mouse 14 days after the first immunization. Blood samples of all groups (6 mice per group) were collected for antibody titer assays on 1, 3, 5, 7, 9, 11, and 13 weeks after the second immunization.

#### 2.5.3. Serum PCV-2-Specific IgG Assay

PCV-2-specific IgG in the serum was determined with enzyme linked immunosorbent assay (ELISA). ELISA kit to detect the antibody against PCV-2 (Wuhankeqian Animal Biological Products Co., Ltd, China) was selected for the serum PCV-2-specific IgG assay, and all operations were carried out in accordance with the kit instructions.

The serum isolated from all groups in a 1 : 1600 dilution was used for PCV-2-specific IgG determination, and 1 : 5000 goat anti-mouse total IgG-HRP conjugate (Jackson Immuno Research, USA) was used as the second antibody. The absorbance was measured at 630 nm using a STAT FAX 2100 microplate reader (AWARENESS, USA).

### 2.6. Statistical Analysis

Data of the optimization of salidroside liposome preparation were analyzed using the Design-Expert program and second-order polynomial equation, and ANOVA of the quadratic regression model, and optimal conditions were shown. Results in this study were presented as mean ± standard errors (S.E.). Statistical comparisons were made by the LSD and Duncan's multiple range test in SPSS (version 19.0), and differences between groups were considered significant if the *P* value was less than 0.05.

## 3. Results

### 3.1. Statistical Analysis and Model Fitting

There were a total of 29 runs for optimizing the four individual parameters in the BBD, and the experimental conditions and the EE of salidroside liposome according to the factorial design were shown in Table 2, which also included the predicted values.

The quadratic model was found to be the fitting model for the EE of salidroside liposome. An empirical relationship expressed by a second-order polynomial equation with interaction terms was fitted between obtained experimental results. The EE obtained in terms of coded factors is calculated as follows:

(3) Y=−221.1667+46.6667X1+19.4000X2+11.5217X3+0.0917X4+13.7500X1X2+3.4000X1X3−0.0550X2X3−0.3750X2X4+0.8200X3X4−540.0000X12−1.5188X22−0.1675X32−5.7125X42.

It was shown that the test chosen quadratic model was highly significant (*P*_model_ < 0.0001) through the analysis of variance of the model from Table 3, and lack of fit was not significant (*P* = 0.2735 > 0.05). Except *X*_1_ *X*_4_ (ammonium sulfate concentration and ethanol injection speed interaction) in the model, the “*P*” values of all quadratics were less than 0.05, indicating that all the models except *X*_1_ *X*_4_ have significant effects on liposome encapsulation efficiency. According to correlation *R* ^2^ = 0.9954 and the correction coefficient of determination *R*_Adj_^2^ = 0.9906, only about 1% of the total variation of liposome encapsulation efficiency could not be explained. In summary, the result showed good fit of the model.

**Figure 1:**
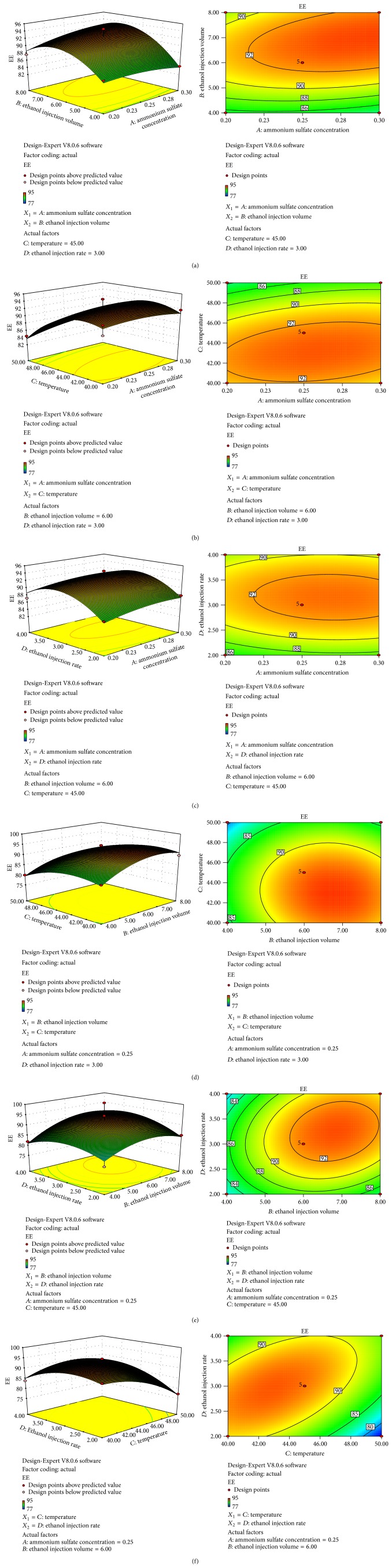
Response surface (3D) and contour plots showing the interaction of different factors on the response *Y*. (a) The interaction of ammonium sulfate concentration and ethanol injection volume, (b) the interaction of ammonium sulfate concentration and temperature, (c) the interaction of ammonium sulfate concentration and ethanol injection rate, (d) the interaction of ethanol injection volume and temperature, (e) the interaction of ethanol injection volume and ethanol injection rate, and (f) the interaction of temperature and ethanol injection rate.

Results of the EE of salidroside liposome affected by ammonium sulfate concentration (mol/L), ethanol injection volume (mL), temperature (°C), and ethanol injection rate (mL/min) were presented in Figure 1. These types of plots showed the effects of two factors on the response at a time and the other factors were maintained at the zero level.

To show the relationship between responses and experiment levels of each variable, the response surface (3D) and contour plots are displayed in Figure 1, respectively. The shapes of the contour plots indicate whether the mutual interactions between the variables are significant or not. Circular contour plot indicates that the interactions between the corresponding variables are negligible, while elliptical contour plot indicates that the interactions between the corresponding variables are significant [18]. The maximum value predicted by the surface was confined in the smallest ellipse in the contour diagram. The elliptical contours were obtained when there was a perfect interaction between the independent variables [19].

The response surface (3D) and contour plot as in Figure 1(a) made EE as a function of ammonium sulfate concentration and ethanol injection volume at fixed temperature and ethanol injection rate. It is indicated that EE rapidly increased with an increase in ethanol injection volume from 4 to 7 mL. In addition, EE increased with an increase in ammonium sulfate concentration from 0.20 to 0.28 mol/L and then reached a plateau region.

Figure 1(b) depicted response surface (3D) and contour plot of the effects of the two variables, namely, the interaction effect of ammonium sulfate concentration and temperature on the EE of salidroside liposome. It indicates that EE gradually increased with an increase in temperature from 40 to 44°C. In addition, EE increased with an increase in ammonium sulfate concentration from 0.20 to 0.28 mol/L and then reached a plateau region. However, the EE was slightly changed after 0.28 mol/L.

The response surface plot (3D) and the contour plot at varying ammonium sulfate concentration and ethanol injection rate at fixed temperature and ethanol injection volume are depicted. As shown in Figure 1(c), the EE was gradually increased before the ethanol injection rate was 3 mL/min. The same variation was found when it came to ammonium sulfate concentration.

Interaction of ethanol volume and temperature was displayed in the 3D response surface plot and the contour plot. According to the results in Figure 1(d), EE gradually increased with an increase in ethanol volume from 4 to 7 mL. In addition, EE increased with an increase in temperature from 40 to 45°C and then reached a plateau region. However, the EE was slightly decreased after 44°C.

Figure 1(e) represented the effects of ethanol volume and ethanol injection rate on the EE of salidroside liposome, while ammonium sulfate concentration and temperature were fixed. Both ethanol volume and ethanol injection rate had significant effects on EE since the contour plot was elliptical. The maximum EE could be achieved when the ethanol injection rate and ethanol volume were within the level range from 3 to 3.5 mL/min and nearly 7 mL.

The interaction effects of temperature and ethanol injection rate were shown in Figure 1(f). The EE gradually increased with an increase in temperature from 40 to 44°C. In addition, EE increased with an increase in ethanol injection rate from 2 to 3 mL/min and then reached a plateau region where the EE was slightly decreased after 3 mL/min.

### 3.2. Verification of the Predictive Mode

To further verify the accuracy of predictions, the model equation for predicting the optimum response values was rechecked under the selected optimal conditions. Additional experiments for reconfirmation were performed under the selected optimal conditions: ammonium sulfate concentration of 0.26 mol/L, ethanol injection volume of 6.5 mL, a temperature of 43°C, and ethanol injection rate of 3 mL/min.

The optimum conditions above were considered to be optimum according to the RSM. In our study, a set of optimum conditions were validated experimentally and the values of the responses using the model equation were predicted. A mean EE value of 94.527 ± 0.682% (*n* = 3) was obtained from practical experiments. The mean EE value was close to the predicted value of 95.422%, which demonstrated the validation of the RSM model.

### 3.3. Results of Encapsulation Efficiency of PCV-2

The results measured by BCA Protein Assay Kit were shown in Table 4. The data showed that when salidroside liposome I PCV-2, salidroside liposome II PCV-2, and salidroside liposome III PCV-2 had the same amount of PCV-2 antigen, 1.1 × 10^7^ TCID_50_/0.2 mL, and all the salidroside liposome PCV-2 could get the maximum encapsulation efficiency of PCV-2 when the volume ratio of salidroside liposome to PCV-2 was 5 : 1. Thus, 5 : 1 volume ratio of salidroside liposome to PCV-2 was selected for the preparation of salidroside liposome PCV-2.

### 3.4. Results of Characteristics of Salidroside Liposome Adjuvant PCV-2 Vaccines

**Figure 2:**
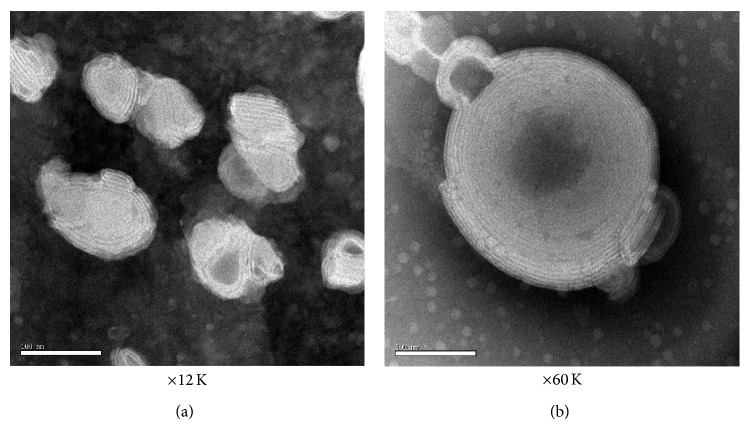
Transmission electron micrograph of salidroside liposome PCV-2.

The particle sizes of three different liposomes were, respectively, 138 ± 22 nm (Salidroside liposome I), 165 ± 18 nm (Salidroside liposome II), and 157 ± 12 nm (Salidroside liposome III) (Table 5). The transmission electron microscope of salidroside liposome PCV-2 was shown in Figure 2. The salidroside liposome PCV-2 presented the homogeneous milk-white and translucent suspension. By transmission electron micrograph, salidroside liposome PCV-2 appeared nearly spherical shape and the monodisperse vesicles. Taken these results together, it was clear that liposomes were of the small particle size and uniform distribution.

### 3.5. In Vitro Release of PCV-2 from Salidroside Liposome PCV-2

**Figure 3:**
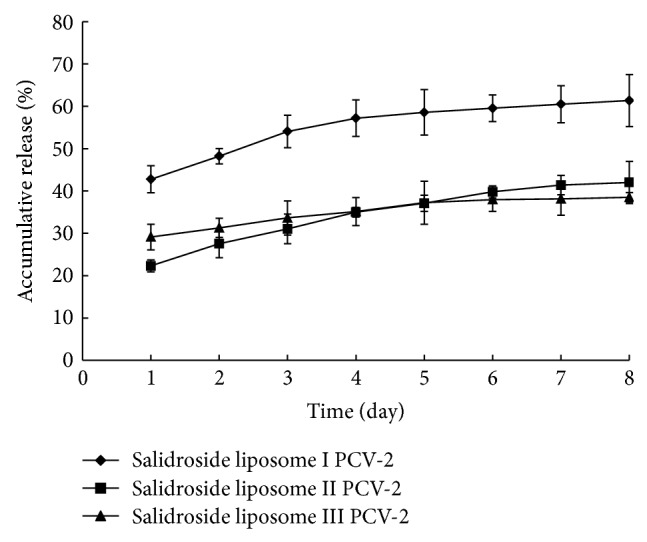
In vitro release profile of salidroside liposome PCV-2.

In vitro, the release of three kinds of salidroside liposome PCV-2 from salidroside liposome was shown in Figure 3. In the early period, the release of salidroside liposome II PCV-2 was the slowest. After 4 days, the release of salidroside liposome II PCV-2 was faster than that of salidroside liposome I PCV-2 and slower than that of salidroside liposome III PCV-2.

### 3.6. Salidroside Liposome as Adjuvant Could Promote the Production of PCV-2-Specific IgG

**Figure 4:**
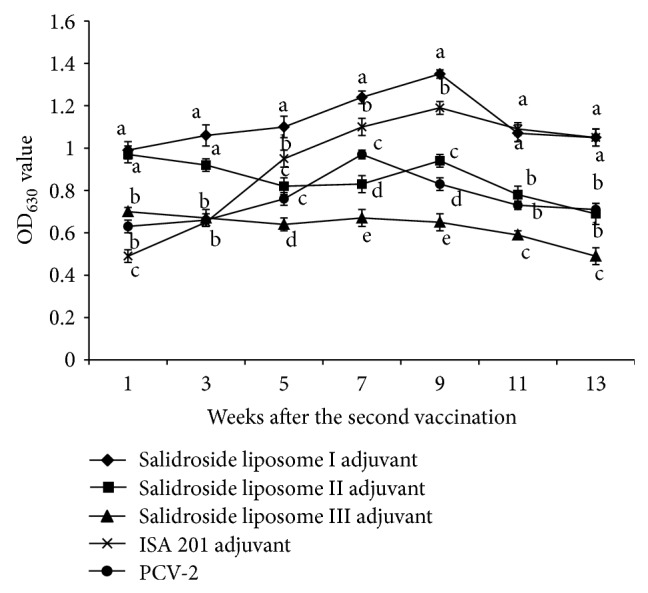
The PCV-2-specific IgG level of different groups after vaccination.

The serum PCV-2-specific IgG levels were shown in Figure 4. The results showed that, on the 1st and 3rd weeks after the second vaccination, the PCV-2-specific IgG levels of salidroside liposome I adjuvant and salidroside liposome II adjuvant groups were significantly higher than those of the other groups (*P* < 0.05). On the 5th, 7th, and 9th weeks after the second vaccination, the PCV-2-specific IgG level of salidroside liposome I adjuvant group was significantly higher than that of the other groups (*P* < 0.05) and the PCV-2-specific IgG level of ISA 201 adjuvant group was significantly higher than those of salidroside liposome II adjuvant and salidroside liposome III adjuvant and nonadjuvant PCV-2 groups (*P* < 0.05). On the 11th and 13th weeks after the second vaccination, the PCV-2-specific IgG levels of salidroside liposome I adjuvant and ISA 201 adjuvant groups were significantly higher than those of the other groups (*P* < 0.05). The results indicated that the salidroside liposome I adjuvant and ISA 201 adjuvant could enhance the production of the PCV-2-specific IgG and the effect of salidroside liposome I adjuvant was superior to the ISA 201 adjuvant.

BALB/c mouse was inoculated twice on days 0 and 14 with PCV-2 encapsulated in three kinds of salidroside liposome adjuvant PCV-2 vaccine, ISA 201 adjuvant PCV-2 vaccine, and PCV-2 vaccine. The levels of PCV-2-specific IgG in serum were measured on weeks 1, 3, 5, 7, 9, 11, and 13 after the second vaccination. Values were mean ± S.E. *n* = 6 mice/group. The superscripts without the same letters near the curves differ significantly (*P* < 0.05) from each other in the same week.

## 4. Discussion

Response surface methodology (RSM) is a combination of statistical and mathematical methods which is helpful for designing experiments. It is convenient for researchers to figure out complex quantitative relationships between parameters and their responses and identify the best combination of responsefactors [20, 21]. The greatest benefit of response surface methodology is that it is able to reduce a number of experiments which are required to evaluate multiple parameters and their interactions [22]. Box-Behnken design (BBD) is one of the most common designs of the response surface methodology because of its rational design and excellent interpretation of experiments [23]. As a consequence, it has been widely used in the optimization of chemical and physical processes.

The 3D response surface plot and contour plot were used to explain the interaction of the variables and to identify the optimum level of each variable to reach a maximum EE yield. The response surfaces plots were presented in Figure 1. Each figure demonstrated the effect of two factors, while the other two figures were fixed at zero level.

It is judged by the shape of the contour plots whether the interactions between the corresponding variables are significant or not. According to the interaction results displayed in Figure 1, these four parameters, ammonium sulfate concentration, ethanol injection volume, temperature, and ethanol injection rate, significantly affect the EE of salidroside. The optimal preparation conditions for the salidroside liposome obtained from the RSM model were as follows: ammonium sulfate concentration (mol/L) was 0.26 mol/L, ethanol injection volume (mL) was 6.5 mL, temperature (°C) was 43°C, and ethanol injection rate (mL/min) was 3 mL/min.

Besides, verification experiment was performed based on the selected optimal conditions and the actual value of EE from the verification experiment was very close to the predicted value. This means that the response surface method is reliable and that the data obtained from the RSM model are trusted.

Porcine circovirus type 2 (PCV-2) is the main virus involved in postweaning multisystemic wasting syndrome (PMWS) and porcine dermatitis and nephropathy syndrome (PDNS) [24–26]. The majority of pig-producing countries suffered significant economic losses because the PCV disease was the increased mortality in severely affected farms and growth retardations in the case of subclinical infection. Therefore, it is of great importance to prevent the PCV-2 infection.

PCV-2 vaccines can provide good immunity effects only if the appropriate adjuvant was added. Nowadays, the main adjuvants used in the PCV-2 vaccine were oil emulsion adjuvants. But these adjuvants are not too much useful for vaccine because of their toxic effects, such as carcinogenic, granuloma, and cyst formation at the site of injection. Therefore, many researches were done to find alternative of the oil emulsion adjuvants and this study investigated the adjuvant activity of salidroside liposome as PCV-2 adjuvant.

The results showed that salidroside liposome as adjuvant could promote the production of PCV-2-specific IgG and maintain higher PCV-2-specific IgG titers for a long time. At early period of immunization (from 1 to 3 weeks), the effect of salidroside liposome I, salidroside liposome II, and salidroside liposome III adjuvants was significantly superior to ISA 201 and nonadjuvant, which indicated that the PCV-2 encapsulated with salidroside liposome could more effectively induce the production of PCV-2-specific IgG. Moreover, significant difference was found between salidroside liposome I and salidroside liposome II adjuvant and ISA 201 adjuvant. From week 5 to week 9, the PCV-2-specific IgG titers of the salidroside liposome I adjuvant were significantly higher than those of ISA 201 adjuvant and those of the other groups. It indicated that humoral immunity response could be boosted when salidroside liposome I served as adjuvant. At later period of immunization (from 11 weeks to 13 weeks), the effect of salidroside liposome I adjuvant was similar to ISA 201 adjuvant, which indicated that salidroside liposome I adjuvant and ISA 201 adjuvant had controlled release. All of these results indicated that salidroside liposome I adjuvant as adjuvant could sustain for a longer effects in the whole vaccination period, which is very important to prevent the PCV-2 infection.

In conclusion, RSM was a kind of valid model to optimize the preparation conditions of salidroside liposome. The optimal preparation conditions for the salidroside liposome were as follows: ammonium sulfate concentration (mol/L) was 0.26 mol/L, ethanol injection volume (mL) was 6.5 mL, temperature (°C) was 43°C, and ethanol injection rate (mL/min) was 3 mL/min. Under these conditions, the experimental EE of salidroside liposome was 94.527 ± 0.68%. Salidroside liposome as adjuvant could rapidly induce the production of PCV-2-specific IgG and sustain higher PCV-2-specific IgG titers for a long time, and the effect of salidroside liposome was superior to ISA 201 adjuvant. These indicated that salidroside liposome could enhance the humoral immunity and had the potential to act as an effective PCV-2 vaccine adjuvant.

## Figures and Tables

**Table 1:** Levels and code of variables chosen for Box-Behnken design.

Factors	Levels and range		
	−1	0	1
*X*_1_: ammonium sulfate concentration (mol/L)	0.20	0.25	0.30
*X*_2_: ethanol injection volume (mL)	4	6	8
*X*_3_: temperature (°C)	40	45	50
*X*_4_: ethanol injection rate (mL/min)	2	3	4

**Table 2:** The design and results of Box-Behnken experiment.

Number	Levels of independent factors				Response: EE (%)	
	*X*_1_	*X*_2_	*X*_3_	*X*_4_	Predicted acquired EE	Practical acquired EE
1	0.30	6	45	4	88.50	88.36
2	0.30	4	45	3	84.10	84.38
3	0.25	6	40	2	91.20	91.18
4	0.25	6	45	3	94.70	84.55
5	0.20	4	45	3	85.70	85.92
6	0.25	6	50	2	76.80	77.27
7	0.25	8	45	4	84.90	94.60
8	0.20	8	45	3	87.30	87.65
9	0.25	6	45	3	94.10	94.60
10	0.30	6	50	3	87.90	87.65
11	0.30	6	40	3	91.30	91.69
12	0.25	4	40	3	85.10	84.63
13	0.30	6	45	2	88.20	87.93
14	0.20	6	40	3	92.20	92.17
15	0.20	6	45	2	86.00	86.21
16	0.25	6	45	3	94.60	94.60
17	0.20	6	50	3	85.40	84.47
18	0.25	8	45	2	85.60	85.12
19	0.25	4	45	2	79.50	79.57
20	0.25	8	40	3	89.40	89.78
21	0.25	8	50	3	82.40	82.95
22	0.30	8	45	3	91.20	91.20
23	0.25	4	45	4	81.80	82.00
24	0.20	6	45	4	87.30	87.22
25	0.25	4	50	3	80.30	80.00
26	0.25	6	40	4	84.20	83.93
27	0.25	6	50	4	86.20	86.40
28	0.25	6	45	3	94.50	94.60
29	0.25	6	45	3	95.10	94.60

**Table 3:** ANOVA for response surface quadratic model.

Source	Sum of squares	df	Mean Square	*F* value	*P* value Prob > *F*	Significance
Model	657.64	14	657.64	216.15	<0.0001	Sig^***^
*X*_1_	4.44	1	4.44	20.43	0.0005	∗∗
*X*_2_	49.21	1	49.21	226.43	<0.0001	∗∗∗
*X*_3_	98.61	1	98.61	453.27	<0.0001	∗∗∗
*X*_4_	2.61	1	2.61	12.03	0.0038	∗∗
*X*_1_ *X*_2_	7.56	1	7.56	34.80	<0.0001	∗∗∗
*X*_1_ *X*_3_	2.89	1	2.89	13.30	0.0026	∗∗
*X*_1_ *X*_4_	0.25	1	0.25	1.15	0.3016	
*X*_2_ *X*_3_	1.21	1	1.21	5.57	0.0333	∗
*X*_2_ *X*_4_	2.25	1	2.25	10.35	0.0062	∗∗
*X*_3_ *X*_4_	67.24	1	67.24	309.40	<0.0001	∗∗∗
*X*_1_^2^	11.82	1	11.82	54.40	<0.0001	∗∗∗
*X*_2_^2^	239.39	1	239.39	1101.54	<0.0001	∗∗∗
*X*_3_^2^	113.74	1	113.74	523.38	<0.0001	∗∗∗
*X*_4_^2^	211.67	1	211.67	974.00	<0.0001	∗∗∗
Residual	3.04	14	0.22			
Lack of fit	2.52	10	0.25	1.94	0.2735	Not sig.
Pure error	0.52	4	0.13			
Cor. total	660.68	28				
*R* ^2^ = 0.9954, *R*_Adj_^2^ = 0.9906, and *R*_Pred_^2^ = 0.9768						

^*^ *P* < 0.05; ^**^ *P* < 0.01; ^***^ *P* < 0.001.

**Table 4:** The results of EE of salidroside liposome PCV-2.

Liposome	The volume ratio	EE	TCID_50_ in liposome/0.2 mL	Total TCID_50_/0.2 mL
Salidroside liposome I PCV-2	2 : 1	10%	2.1 × 10^5^	2.1 × 10^6^
	3 : 1	30%	4.7 × 10^5^	1.6 × 10^6^
	4 : 1	40%	5.1 × 10^5^	1.3 × 10^6^
	5 : 1	60%	6.3 × 10^5^	1.1 × 10^6^
	6 : 1	55%	5.0 × 10^5^	9.0 × 10^5^

Salidroside liposome II PCV-2	2 : 1	8%	3.3 × 10^5^	4.2 × 10^6^
	3 : 1	22%	6.9 × 10^5^	3.2 × 10^6^
	4 : 1	44%	7.0 × 10^5^	1.6 × 10^6^
	5 : 1	80%	8.4 × 10^5^	1.1 × 10^6^
	6 : 1	76%	6.9 × 10^5^	9.0 × 10^5^

Salidroside liposome III PCV-2	2 : 1	10%	4.2 × 10^5^	4.2 × 10^6^
	3 : 1	30%	9.5 × 10^5^	3.2 × 10^6^
	4 : 1	50%	7.9 × 10^5^	1.6 × 10^6^
	5 : 1	75%	7.9 × 10^5^	1.1 × 10^6^
	6 : 1	70%	6.3 × 10^5^	9.0 × 10^5^

**Table 5:** The characteristics of three kinds of liposomes.

Liposome	Phospholipids	Salidroside	PCV-2	EE	Size (nm)
	(mg/mL)	(mg/mL)	(TCID_50_/0.2 mL)		
Salidroside liposome I PCV-2	30	0.5	1.1 × 10^6^	60%	138 ± 22
Salidroside liposome II PCV-2	60	0.5	1.1 × 10^6^	80%	165 ± 18
Salidroside liposome III PCV-2	90	0.5	1.1 × 10^6^	75%	157 ± 12

## Abbreviations

EE: Encapsulation efficiency
RSM: Response surface methodology
PCV-2: Porcine circovirus type 2 virus
PBS: Phosphate buffered saline
BBD: Box-Behnken design
ELISA: Enzyme linked immunosorbent assay
PMWS: Postweaning multisystemic wasting syndrome
PDNS: Porcine dermatitis and nephropathy syndrome
BALB/c mice: An albino, laboratory-bred strain of the house mouse
MWCO: Molecular weight cutoff
BCA: Bicinchoninic acid
IgG: Immunoglobulin G
ANOVA: Analysis of variance
S.E.: Standard error
LSD: Least significant difference.
